# Deep Learning for Non-Invasive Diagnosis of Nutrient Deficiencies in Sugar Beet Using RGB Images

**DOI:** 10.3390/s20205893

**Published:** 2020-10-18

**Authors:** Jinhui Yi, Lukas Krusenbaum, Paula Unger, Hubert Hüging, Sabine J. Seidel, Gabriel Schaaf, Juergen Gall

**Affiliations:** 1 Computer Vision Group, Institute of Computer Science III, University of Bonn, Endenicher Allee 19a, 53115 Bonn, Germany; gall@iai.uni-bonn.de; 2 Plant Nutrition Group, Institute of Crop Science and Resource Conservation, University of Bonn, Karlrobert-Kreiten-Strasse 13, 53115 Bonn, Germany; s7lukrus@uni-bonn.de (L.K.); paulaungera@gmail.com (P.U.); gabriel.schaaf@uni-bonn.de (G.S.); 3 Crop Science Group, Institute of Crop Science and Resource Conservation, University of Bonn, Katzenburgweg 5, 53115 Bonn, Germany; h.hueging@uni-bonn.de (H.H.); Sabine.Seidel@uni-bonn.de (S.J.S.)

**Keywords:** nutrient deficiencies, sugar beet, deep learning, nitrogen, phosphorous, potassium, liming

## Abstract

In order to enable timely actions to prevent major losses of crops caused by lack of nutrients and, hence, increase the potential yield throughout the growing season while at the same time prevent excess fertilization with detrimental environmental consequences, early, non-invasive, and on-site detection of nutrient deficiency is required. Current non-invasive methods for assessing the nutrient status of crops deal in most cases with nitrogen (N) deficiency only and optical sensors to diagnose N deficiency, such as chlorophyll meters or canopy reflectance sensors, do not monitor N, but instead measure changes in leaf spectral properties that may or may not be caused by N deficiency. In this work, we study how well nutrient deficiency symptoms can be recognized in RGB images of sugar beets. To this end, we collected the Deep Nutrient Deficiency for Sugar Beet (DND-SB) dataset, which contains 5648 images of sugar beets growing on a long-term fertilizer experiment with nutrient deficiency plots comprising N, phosphorous (P), and potassium (K) deficiency, as well as the omission of liming (Ca), full fertilization, and no fertilization at all. We use the dataset to analyse the performance of five convolutional neural networks for recognizing nutrient deficiency symptoms and discuss their limitations.

## 1. Introduction

The detection of nutrient deficiencies refers to the task of recognizing nutrient limitations of crops, such as nitrogen (N), phosphorous (P) and potassium (K) deficiency. Plants require 14 essential mineral elements to grow and to complete their life cycle [1,2]. The availability for plant growth is distinct for each element and is strongly influenced by the genetics of the crop (i.e., by the type of crop species and variety), by soil properties, including soil pH, and by the type of applied fertilizer [1,2,3]. Soil acidification, as a consequence of a number of factors, such as acidic precipitation, nutrient removal by harvest, fertilization with ammonia (NH_4_^+^), urea or elemental sulfur (S), and mineralization of organic matter, can result in nutrient deficiencies as well as toxicities of certain nutrients and non-essential elements. As a consequence, liming, i.e., the addition of liming materials containing calcium carbonate (CaCO_3_) or calcium oxide (CaO), is important for ameliorating soil acidity, especially in unbuffered soils, in order to prevent harmful nutrient imbalances [1,4,5,6]. Early recognition of nutrient deficiencies is crucial in practice to allow for timely intervention and avoid irreversible losses, because symptoms of nutrient deficiencies visible to the human eyes often appear when plants are already severely damaged.

Currently, non-invasive technologies, such as chlorophyll meters or canopy reflectance sensors, are widely adopted. However, these optical sensors do not directly monitor N content in plant tissues but instead measure changes in leaf spectral properties that might be induced by N deficiency [7,8,9,10]. Often, they work quite well, since N deficiency is prevalent in many agricultural systems. However, drought stress, damages caused by frost or herbicides, deficiencies in other nutrients, as well as symptoms that are caused by pathogens, can easily be interpreted as N-deficiency by current sensor technology with detrimental consequences for crop management, i.e., poor yield and excess N-application with severe environmental consequences [7,8]. Other non-invasive methods that are based on images also play a significant role in monitoring nutrient status. NDVI is a simple indicator used to identify vegetated areas and their conditions (including nutrient status) with remote sensing data. However, to our knowledge, NDVI does not allow for accurately discriminating between N, P, K, and other nutrient deficiencies. Besides, hyper-spectral imaging was recently shown to enable the diagnosis of nutrient deficiencies in soy and maize from a large number of different macro- and micro-nutrient deficiencies and additionally drought stress with variable accuracy [11]. However, hyper-spectral imaging requires expensive equipment and controlled light conditions, which are not practical to the farmers. Additionally, it lacks spatial resolution with the disadvantage that fine patterns, such as interveinal chlorosis, rolling, or curling of leaves, are not accurately resolved. Likewise, destructive nutrient analyses of plant samples are time consuming, expensive, do not allow for the monitoring the nutrient status in real time and even do not guarantee the unambiguous identification of the growth-limiting nutrient particular when several nutrients are in imbalance, such as it is often the case when crops are cultivated on strongly acidic or alkaline soils [1]. Therefore, we aim to look for a cheaper and more practical method to diagnose nutrient deficiencies.

Over the last few years, deep-learning-based computer vision problems have been extensively studied thanks to the rise of convolutional neural networks (CNNs) [12,13,14,15,16,17,18]. Driven by tremendous economic potential, deep learning is adopted for agricultural applications, such as aerial image semantic segmentation [19], plant disease recognition [20,21], or crop classification [22,23], with the help of RGB images. However, the datasets they worked on only contain images of leaves or aerial images and none of them tried to detect nutrient deficiencies for sugar beets. For an early, non-invasive, and on-site detection of nutrient deficiencies, we used RGB images of entire plants in a sugar beet field with nutrient deficiency plots that were established by decades of nutrient omission at the long-term fertilizer experiment Dikopshof [24]. In this work, we investigate, for the first time, how well convolutional neural networks are able to diagnose N, P and K deficiencies as well as nutrient imbalances caused by soil acidification in sugar beet throughout the growing season, which is an important step to enable appropriate nutrient control and increase yield potential at minimal economic and environmental cost.

**Figure 1:**
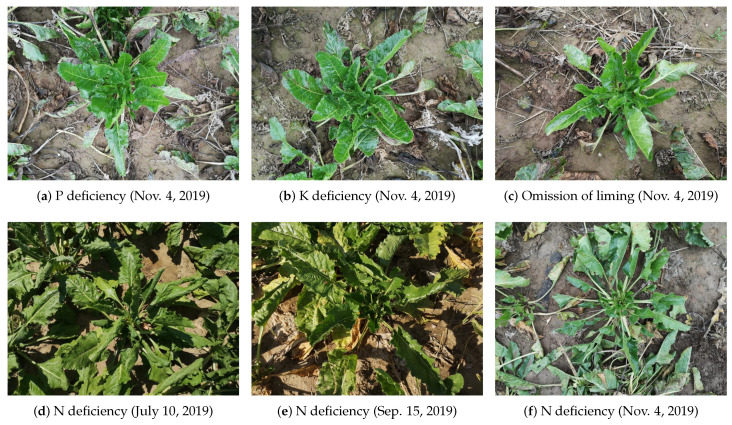
Example images. (**a**–**c**) Sugar beets with similar visual appearance but different nutrient deficiencies, indicating low inter-class visual variation. (**d**–**f**) Sugar beets with totally different visual appearance but all subjected to N deficiency, indicating high intra-class visual variation.

When compared to typical classification problems, classifying the nutrient status of crops is a challenging task, even for human experts, as images with different nutrient deficiencies may have similar visual appearances, while images with the same nutrient limitation may show great variations, as shown in Figure 1. The first row illustrates three sugar beet plants with similar viewpoint, brightness, and background, but with different nutrient deficiencies, namely P deficiency, K deficiency, and omission of liming, respectively. The visual appearances of these images are very similar, demonstrating a case of low visual variation among classes. In comparison, the second row depicts three images with the same deficiency, but different viewpoint, brightness, and background. The visual variation within N deficiency is much larger than the variation among classes in the first row. It is worth noting that these images were taken at different dates, which also introduced enormous variation due to changing environmental conditions and crop development stages.

We present DND-SB (Deep Nutrient Deficiency for Sugar Beet), a high-quality RGB dataset of sugar beet images to advance studies for the detection of nutrient deficiencies, in order to encourage research on this challenging task. The images were taken in seven plots (plot size: 21 × 15 m) in 2019 during the whole growth period of sugar beet at the long-term fertilizer experiment Dikopshof that was established in 1904. The experiment comprises plots that received no N, no P, no K, or were not subjected to liming, plots which were not fertilized at all (control) and optimally fertilized plots (with or without manure). DND-SB is the first dataset that allows forto evaluate approaches for diagnosing different types of nutrient deficiencies in sugar beets. For evaluation, we analyzed the performance of five convolutional neural networks for extracting distinctive features that differentiate nutrient deficiencies under different experimental settings. Our main contributions are summarized, as follows:

- we introduce a high quality RGB image dataset named DND-SB for nutrient deficiencies classfication in sugar beets;
- we present a systematic evaluation of recent CNN-based architectures on the proposed dataset to encourage further research; and,
- we highlight the distinct impact of crop development stages on the classification results.

## 2. Related Work

Deep-learning-based frameworks have shown their astonishing performance in a wide range of tasks over the last few years, including image classification [12,13,14,15,16,17,18]. Driven by promising performance and tremendous economic potential, deep learning were recently adopted in crop management, including disease detection [20,21], weed detection [25], species recognition [22,23], and aerial image semantic segmentation [19]. To evaluate the overall performance of deep learning in these agricultural scenarios, Kamilaris and Prenafeta-Boldú [26] conducted a survey of 40 relevant papers using deep learning models, including CNN-based models (AlexNet, VGG, ResNet, DenseNet), RNNs (Long Short-Term Memory, Differential Recurrent Neural Network), and author-customized networks.

Most of previous works conducted experiments on analyzing and detecting certain kinds of disease in plants. Sladojevic et al. [20] recognized 13 types of plant diseases by applying CNN-based models to leaf images and achieved an averaged precision of 96.3%. Hallau et al. [21] used smartphones and in-house servers to identify sugar beet diseases with support vector machines (SVM). Ramcharan et al. [27] detected foliar symptoms of diseases in cassava with a CNN model that was deployed on a mobile device.

Instead of monitoring and detecting diseases, recent studies have focused on analyzing nutrient deficiency on the growth of plants and/or crops. Han and Watchareeruetai [28] detected seven nutrient treatments with ResNet-50 in a dataset containing 4088 images of black gram (*Vigna mungo*) and reported a test accuracy of 65.44%. Note that our datasets are similar in terms of number of images and treatments, but their reported accuracy is not promising, possibly due to the fact that the dataset they used only contains images of leaves, where pathological symptoms are easily misinterpreted. Tran et al. [29] classified three nutrient deficiencies for 571 tomato images using an ensemble of an Inception-ResNet and an autoencoder, achieving an accuracy of 91% in the test set. However, the dataset that they used is small-scale and only contains treatments of N, K, Ca.

The symptoms of nutrient deficiencies of sugar beet include yellowing of older leaves (typical for N deficiency), other discoloration of the whole leaves, brown veining, stunted greening (typical for P deficiency), wilting, and necrotic regions at the leaf margins and between veins also called leaf scorch (typical for K deficiency), or crinkling, downward cupping in younger leaf blades, and irreversible damage of tip meristems (typical for Ca deficiency) [30]. Additionally, the plant size may be decreased and yield may be reduced. However, most of these previous datasets only contain images of leaves and none of them are dedicated to nutrient deficiency detection in sugar beets. As symptoms of leaves that are caused by different nutrient deficiencies are prone to be recognized as nitrogen deficiency, it is of interest to recognize and predict deficiencies based on the whole plants that might offer global context instead of local patterns. For this purpose, we have collected a dataset which consists of 5648 RGB images of sugar beets in fields under controlled conditions. Besides, previous works did not consider the domain discrepancy problem that was introduced by crop stages. Hence, we highlighted the effect of crop stages on the detection performance while applying CNN models.

## 3. Deep Nutrient Deficiency for Sugar Beet (DND-SB) Dataset

**Figure 2:**
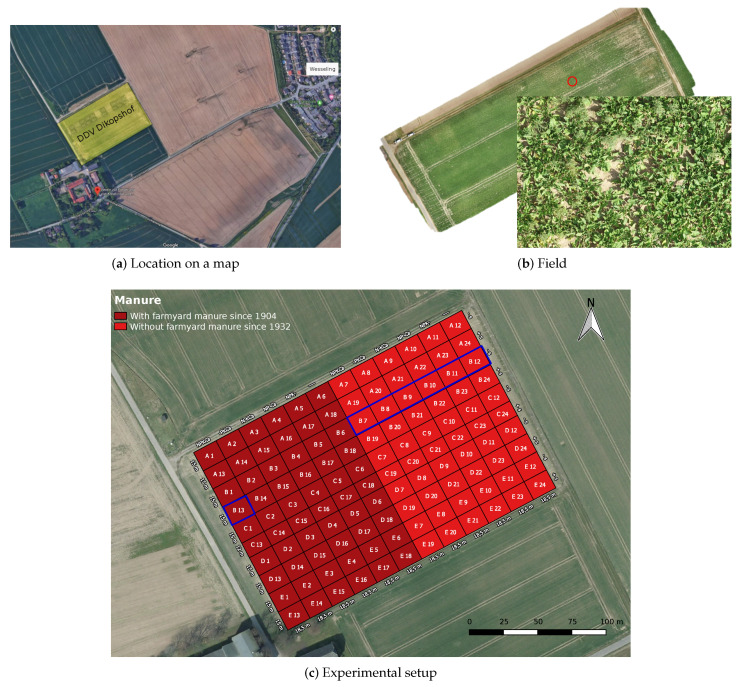
Aerial views of (**a**) the field (**b**) the field at the long-term fertilizer experiment (LTFE) Dikopshof, Wesseling. (**c**) Experimental setup. +s stands for supplemental fertilization. The considered plots (B7-B13) in stripe B are marked with a blue frame. Best viewed in electronic form.

The proposed Deep Nutrient Deficiency for Sugar Beet (DND-SB) dataset consists of 5648 RGB images of sugar beet captured over the whole growth period in 2019 at the long term fertilizer experiment (LTFE) Dikopshof near Bonn, where the field is illustrated in Figure 2. The sugar beet (*Beta vulgaris* variety BTS 7300 N from BETASEED) was sown on April 8 and the harvest started in November 2019. The growth period in 2019 was characterized by a prolonged dry spell in June and July, leading to visible drought stress symptoms, such as wilting. We first provide additional information regarding the experimental site in Section 3.1 and then discuss how the images have been collected in Section 3.2. In Section 4, we provide an additional analysis of the soil and shoots.

### 3.1. Experimental Site

The long-term fertilizer field experiment Dikopshof was established in 1904 in Wesseling near Cologne, Germany (GPS coordinates: 50°48′21″ N, 6°59′9″ E, altitude: 61 m), located at the intermediate strath terrace of the Rhine river. This long-term field experiment is the tenth oldest long-term field experiment in the world. The Atlantic climate with mild winters and summers results in a mean annual temperature of 10.1 °C and a mean annual precipitation of 630 mm. The general soil type is classified as a Haplic Luvisol that is derived from loess above sand [31]. The clay-depleted topsoil horizon (Al) is concordant with the plowed Ap horizon (0–30 cm), followed by an illuvial Bt horizon down to about 80 cm, which is characterized by an increase in clay content. The subsequent cambic horizon is 20 cm thick, followed by a layer of loess of about 60 cm that is present until the sand, and gravel layers start beyond 160 cm depth ([31] and own observations). The experimental design can be described as a non-randomized block design and comprises five stripes and six main treatments: NPKCa, _PKCa, N_KCa, NP_Ca, NPK_ (_ stands for the omission of the corresponding nutrient), and no fertilizer applied [24]. The size of each of the plot is 15 × 18.5 m with a core plot of 9 × 10 m. In this study, seven treatments in strip B cultivated with sugar beet were focused (Figure 2c). One can assume a lack of the specific nutrient due to the omission of the specific nutrient and lime for many decades. To quantify the nutrient deficiency, soil nutrient and shoot nutrient analysis were conducted at the same plots on representative locations in case of soil samples or plants in case of shoot nutrients. The images taken by the camera (protocol see below) as well as the soil samples and shoot samples for analysis of soil and shoot nutrient concentrations were all sampled in the core plot of the same seven plots (Figure 2c). Thus, the sampling points for the image acquisition and the sampling points for performing the soil and shoot nutrient analysis are identical.

### 3.2. Image Acquisition Protocol

The images were annotated with seven types of fertilizer treatments: unfertilized, _PKCa, N_KCa, NP_Ca, NPK_, NPKCa, NPKCa+m+s. N, P, K refer to the respective nutrients while Ca refers to liming. In this nomenclature, _ stands for the omission of the corresponding nutrient treatment or liming, while m+s indicates application of supplementary mineral fertilizer and manure. More specifically, these images were taken in seven plots (B7–B13 in Figure 2c), each of which was subjected to a type of nutrient treatment. For example, an image with the label of “_PKCa” indicates that it was taken in a plot that received no N (plot B8). All of the RGB images with the size of 7296 × 5472 were captured by a Huawei P20 Pro smartphone with triple camera from Leica under natural lighting condition. Example images are shown in Figure 1. Note that all of the images were taken under different conditions in terms of height, viewpoint, light, and weather to reflect realistic conditions as they occur in practice.

We use the dataset to systematically evaluate how accurate recent CNN-based architectures, which are described in Section 5, recognize each nutrient deficiency and how crop development stages affect the performance. Because a model’s ability to recognize a pattern during inference is determined by the frequency at which the model observes a pattern during training, it is crucial to understand the sample distribution for each annotation category and for each date. Therefore, the statistics of the DND-SB dataset is presented in Table 1. Although most annotations have a similar amount of images, there is a small imbalance of the sample distribution among different dates.

In most of our experiments, we randomly sampled 70% of the images as a training set and the rest as a test set, which resulted in 4006/1642 train/test images. We also split the images into training set and test set based on the dates they were taken while analyzing the effect of crop development stages, e.g., images from the first seven dates as training set and the rest as test set. The dataset is available at https://github.com/jh-yi/DND-SB.

## 4. Soil and Plant Nutrient Analyses

### 4.1. Soil Nutrient Analyses

Soil samples (one per treatment and date) were taken from the topsoil (about 0–25 cm depth) and directly frozen on four dates in order to validate the specific nutrient deficiency in each plot, as shown in Table 2. The frozen soil samples were thawed and the mineral nitrogen concentration ($N_{\min}$) was determined. A Skalar Continuous Flow Analyzer was used for mineral N concentration (NO_3_^−^ and NH^4^+) analysis. The concentrations of soil P and K ($P_{\mathrm{CAL}}$ and $K_{\mathrm{CAL}}$) available to plants were determined with a calcium-acetate-lactate extract. Additionally, the pH value of the respective soil samples was determined (CaCl solution with a pH Meter—Multi 3630 IDS WTW and Sentix 940P electrode). As expected, withholding N fertilizer (_PKCa treatment) resulted in a strong depletion in mineral N in all time points as compared to the NPKCa control. Likewise, withholding P or K resulted in a strong reduction of calcium-acetate-lactate extractable P and K, respectively, in all analyzed time points. Likewise, the no liming control (NPK_) and no fertilizer treatment resulted in a reduction in soil pH, as expected. We further observe that omission of P and K results in increased soil mineral N. This is not surprising, as stunted plants (due to P or K deficiency) are expected to have a reduced demand for N, hence N accumulates in the topsoil.

### 4.2. Plant Nutrient Analyses

**Figure 3:**
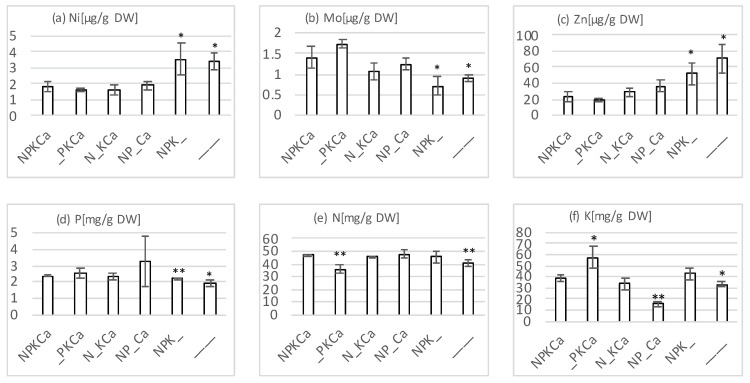
Nutrient contents of young sugar beet leaf blades at the LTFE Dikopshof Wesseling harvested on 26 August 2019. Three young leaves from different plants grown under fertilization treatments as indicated were dried for seven days at 60 °C. Approximately 7 mg of ground plant material was subjected to N determination by an elemental analyzer while approximately 500 mg were digested by nitric acid and then subjected to either K-determination by flame photometry or ICP-OES measurements (Ni, Mo, Zn, and P). Error bars indicate standard deviation. Each treatment was tested against the NPKCa-treatment using an unpaired, two-sided *t*-Test (n = 3). Significance is indicated as * for *p* = 0.05 and ** for *p* = 0.01.

**Figure 4:**
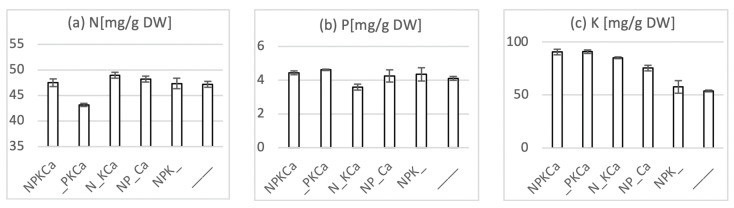
Nutrient contents of young sugar beet leaf blades at the LTFE Dikopshof Wesseling harvested on 13 June 2019. Pooled samples of shoots from five plants grown under fertilization treatments, as indicated were dried for seven days at 60 °C. Approximately 7 mg of ground plant material was subjected to N determination by an elemental analyzer. Three measurements were performed on the same sample. Three independent digestions of approximately 500 mg were generated by nitric acid digestion and then subjected to either K-determination by flame photometry or P-determination by ICP-OES. Error bars indicate standard deviation. Note that, since samples were pooled, standard deviation only accounts for uncertainty in measurement.

Sugar beet leaves of three randomly chosen plants within the core plot were harvested on 26 August 2019 in order to investigate whether the dry spell compromised the desired nutrient deficiencies in the respective fertilizer treatments. Shoots of five randomly chosen plants within the core plot were harvested on 13 June 2019. Samples that were harvested on June 13 were pooled into a single sample. To this end, plant material was dried, ground and digested by nitric acid (as described in [32]). The quantification of potassium was done by flame photometry (ELEX 6361, Eppendorf AG, Hamburg, Germany). Quantification of phosphorus, nickel, zinc and molybdenum was done by Inductively Coupled Plasma Optical Emission Spectrometry (ICP-OES) (Horiba John Yvon Model 75 Plus, Unterhaching, Germany). Total N was determined with the help of an elemental analyzer (Euro Vector CHNS-O, HEKAtech GmbH, Wegberg, Germany). Data from the elemental analyzer were calculated into peak areas by the CallidusTM software 4, providing quantitative results while using reference material as a calibrating standard. For samples that were harvested on 26 August, one measurement was performed for each sample and each treatment was tested against the NPKCa-treatment using an unpaired, two-sided t-Test (n = 3). For pooled samples that were harvested on 13 June, digestions were done as technical triplicates. For P- and K-determination, a single measurement was performed for each digestion while for N determination three technical replicates were made from dried and ground plant samples. The results for samples harvested on 26 August are shown in Figure 3. As expected, a significant reduction in total N and K was observed in the _PKCa and NP_Ca treatment, respectively. Both of the elements were also reduced in the unfertilized plot (Figure 3e–f). In contrast, no reduction in P content was detected in leaves of plants grown under the N_KCa treatment (Figure 3d). A 19% lower P content of leaves harvested on 13 June 2019 (Figure 4b, i.e., before the dry spell) in treatment N_KCa compared to NPKCa suggests that the drought event attenuated P-deficiency. This was not unexpected, since nutrient demand is reduced when growth is compromised, since it provides plants with more time and opportunities to mobilize P from less available soil fractions. Something similar has been observed before for white clover plants [33]. We hypothesized that P deficiency experienced at early growth stages by plants grown at the N_KCa plot might have imprinted a phenotypic memory even when a decrease in P was not detected any more at later stages due to growth retardation. Therefore, we decided to continue taking images of these plants, also beyond the drought spell events.

In the NPK_ (no liming) as well as the unfertilized treatment, sugar beet leaves displayed an increase in Zinc- and Nickel-content, as well as a decrease in Molybdenum-content (Figure 3a–c). This observation is in agreement with an alkalizing effect of lime, since a decrease in soil pH (no liming) is known to increase cation (Ni^2^+, Zn^2^+) solubility and, hence, availability while it reduces the solubility and therefore uptake of anions such as MoO_4_^2^− [1].

Similarly, a reduction of N and K was also observed in the sample that was harvested on 13 June (Figure 4a,c). Interestingly, plants harvested from the unfertilized plot displayed a 40% reduction in K-content compared to the NPKCa-treatment, while only minor reductions for P and N were observed. This suggests that, under these conditions, K appeared to represent the growth limiting nutrient in agreement with the high demand of sugar beet for this nutrient reported before at DDV Dikopshof [24].

## 5. Baselines

### 5.1. Convolutional Network Architectures

In this section, we briefly describe the CNN architectures AlexNet [14], VGG [15], ResNet [16], DenseNet [17], and SqueezeNet [18] that we will use for evaluation. We used models that have been pre-trained on ImageNet [34], a large-scale dataset for image classification. Due to its large amount of images and large number of classes, models that perform well in ImageNet can be successfully adapted to a variety of target datasets by transferring the general-purpose features [35] that they learned. For comparison, we also trained each model from scratch in order to validate the effectiveness of pre-training.

**Figure 5:**
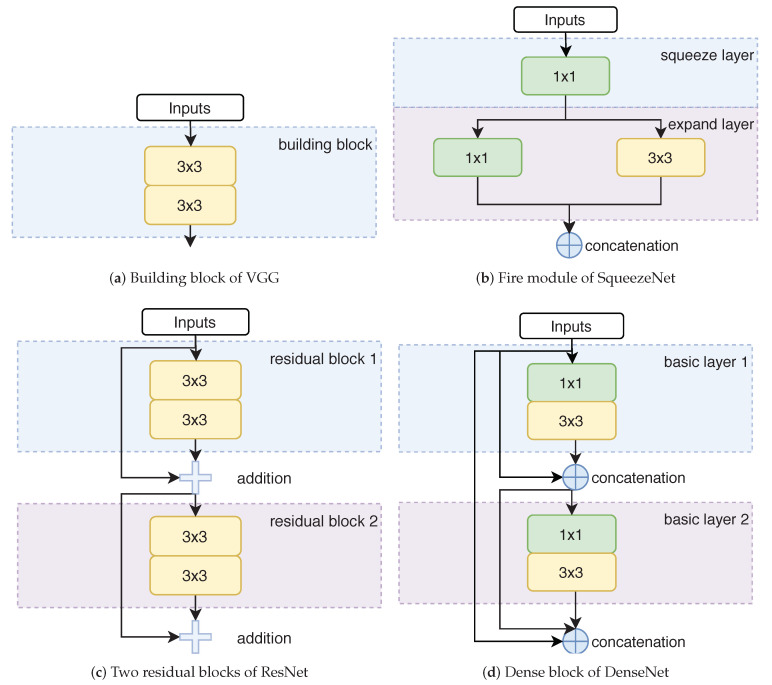
Comparison of basic building blocks of (**a**) the VGG architecture, (**b**) the SqueezeNet architecture, (**c**) the ResNet architecture, (**d**) the DenseNet architecture. For better comparison two residual blocks are illustrated. A n×n block indicates a convolutional layer with the kernel size of n×n.

AlexNet [14] is a small network that uses several techniques for effective and efficient training, including dropout [36], data augmentation (random crop, horizontal flip and random RGB color shift) and ReLU non-linearity [37]. It consists of five convolutional layers followed by three fully connected layers. The kernel sizes are 11 × 11 and 5 × 5 in the first and second convolutional layer, and 3 × 3 in the other convolutional layers. When compared to AlexNet, VGG [15] uses stacks of small 3 × 3 convolutions instead of large convolutions, as shown in Figure 5a, because a sequence of 3 × 3 kernels have the same receptive field as a single larger kernel while saving parameters. However, the network also contains more layers and, therefore, has more parameters, as shown in Table 3. The large number of parameters, however, makes it prone to overfitting on the training data, which, hence, deteriorates its generalization ability during inference.

The ResNet architecture [16] uses residual blocks with shortcut connections, as shown in Figure 5c, which allow for training deeper models, since they avoid the vanishing gradient problem. Specifically, gradients from the outputs of each block can jump over all intermediate layers via the shortcut connections and reach the inputs of the block without being diminished. By stacking these residual blocks, ResNet can be trained with 101 or more layers, which are much more when compared to VGG with less than 20 layers. Despite the deeper architecture, ResNet has much less parameters than VGG, since it utilizes 1 × 1 convolutions to reduce the number of channels inside each residual block. Generally, ResNet consists of a 7 × 7 convolutional layer, followed by a sequence of residual blocks with kernel size of 3 × 3 or 1 × 1 and a fully connected layer.

Inspired by ResNet, DenseNet [17] further increases the number of shortcut connections. Specifically, each dense block contains multiple shortcut connections, as each layer is connected to all subsequent layers inside of this block, as illustrated in Figure 5d. In comparison, there is a single shortcut connection that connects the input and the last layer in a residual block. Furthermore, DenseNet combines features through concatenations instead of addition in ResNet, as concatenations help to increase the variation of features for subsequent layers. Although the concatenation operation helps each layer to receive a ‘collective knowledge’ from all preceding layers in a dense block, it would dramatically increase the channels of features, resulting in much higher computational complexity. For the purpose of compressing redundant features, a basic layer (a 1 × 1 convolution followed by a 3 × 3 convolution) is used. Hence, the layers that connect each other within a dense block are basic layers instead of a single convolutional layer. Because of this design choice, DenseNet requires less parameters than ResNet for the same number of layers. Similarly, DenseNet consists of a 7 × 7 convolutional layer followed by a sequence of dense blocks and transition layers (1 × 1 convolution, followed by 2 × 2 average pooling) and a fully connected layer.

Because the aforementioned models can be computationally too demanding for resource-limited platforms (e.g., mobile devices or embedded systems) due to millions of parameters, we also evaluate SqueezeNet [18] that uses the fire module as a basic building block, which comprises a squeeze layer (consisting of purely 1 × 1 filters) followed by an expand layer (consisting of a combination of 1 × 1 and 3 × 3 filters), as shown in Figure 5b. Most of the filters have a kernel size of 1 × 1 instead of 3 × 3 (the ratio can be controlled in the expand layer), as the latter has nine times more parameters than the former. Besides, the number of parameters in a convolution layer is calculated as ($C_{in}*C_{out}*K*K$), where $C_{in}$ is the number of input channels, $C_{out}$ is the number of output channels, which is also equal to the number of filters, and *K* is the kernel size of the filters. The 1 × 1 convolutions in the squeeze layer result in a strong decrease in the number of input channels to the expand layer, which dramatically decreases the number of parameters. In general, SqueezeNet consists of a 7 × 7 convolutional layer, followed by a sequence of fire modules and a 1 × 1 convolutional layer. It is also worth noting that there is no fully-connected layers in SqueezeNet, which is inspired by the NiN architecture [38]. The reason for this is that fully-connected layers require much more parameters than convolutional layers.

### 5.2. Details of Training

**Figure 6:**
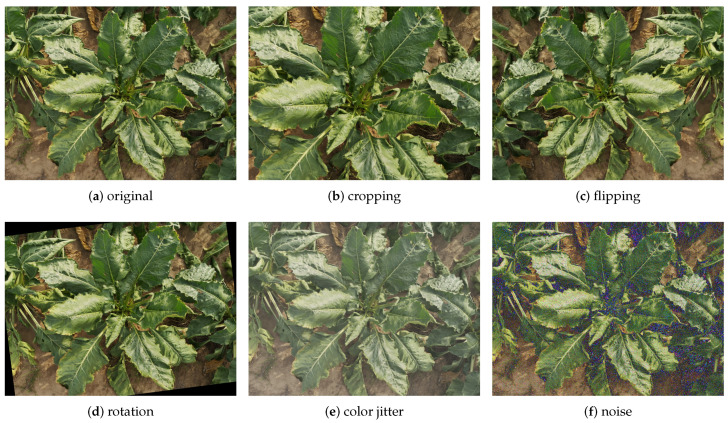
Example images after applying data augmentation techniques. Note that the noise value is exaggerated for better visualization.

If not specifically pointed out, the default hyper-parameters in our experiments are, as follows: The original image was resized to 592 × 592 with square padding and normalized with a mean value of [0.485, 0.456, 0.406] and a standard deviation of [0.229, 0.224, 0.225] calculated from ImageNet. For data augmentation, we apply default random rotations and randomly flip the images. The purpose of data augmentation is to increase the variability of the training images in a dataset, which helps the trained model to generalize better to images that were obtained from different scenarios. We also investigate other forms of data augmentation like cropping, color jittering or adding noise, as shown in Figure 6.

If we trained the networks from scratch, then the parameters of the neural networks were randomly initialized using the Xavier algorithm [39], otherwise we used models that were pre-trained on ImageNet. We then trained each model for 50 epochs with a batch size of 4. We used stochastic gradient descent (SGD) with an initial learning rate of $10^{-3}$, where the momentum and weight decay were set as 0.9 and $10^{-4}$, respectively. The learning rate was reduced by a factor of 0.1 when no improvement was observed for three epochs, and the training process stopped early when the learning rate was smaller than $10^{-7}$. All of the experiments were conducted with a single Nvidia GeForce GTX 1080Ti. For evaluation we report the top-1 accuracy metric on the test set, which denotes whether the predicted category with the highest confidence matches the ground truth category.

## 6. Experimental Results

### 6.1. Evaluation of CNNs for Nutrient Deficiency Detection

We analyzed and compared five different network architectures in terms of accuracy, training time, test time, and number of parameters with and without pre-trained models in order to evaluate how accurate RGB-image-based nutrient deficiency detection can be. For each network architecture, we performed five experimental trials with the same hyper-parameter configuration where the only difference was the random seed. The results are shown in Table 3, where each number is the average over five trails.

The table shows that all of the trained models achieve an accuracy over 87% for classifying nutrient deficiencies in sugar beet. The networks ResNet-101 and DenseNet-161 outperform the other models by a large margin, mainly due to their gradient-stabilizing shortcut connections. The best performance is achieved by DenseNet-161 with an accuracy of 98.4%, which is an increase of 3.5% over ResNet-101. SqueezeNet has the fewest parameters; hence, it is more suitable for resource-limited platforms, but it comes at the cost of a much lower accuracy as compared to DenseNet-161.

While training with pre-trained models, none of the pre-trained layers were frozen, as the DND-SB dataset differs a lot from ImageNet and the models need to adapt to our dataset. Pre-training is essential and the pre-trained models outperform their counterparts trained from scratch in terms of accuracy and training time, as we can see from Table 3. Although [40] has reported that pre-training can only help to speed up convergence, but it not necessarily improves the accuracy if the target dataset is large (e.g., >10 k images), pre-training is crucial for classifying nutrient deficiencies as acquiring data in the field is very difficult and training data will always be a limiting factor. It is interesting to note that only for DenseNet-161 the accuracy without pre-training drops by less than 10% and that the number of parameters are not a good indicator how well a network performs if it is trained from scratch.

While ResNet-101 and DenseNet-161 infer the nutrient deficiencies from a single image in equal or less than 0.5 s, which corresponds to 20–23 images per second, SqueezeNet and AlexNet analyze 90 and 111 images per second, respectively. Therefore, the networks provide different trade-offs between accuracy, memory footprint, and processing time. While DenseNet-161 achieves the highest accuracy, AlexNet is the fastest network and SqueezeNet has the lowest memory footprint. Only ResNet-101 is, for all measurements, outperformed by DenseNet-161.

### 6.2. Impact of Data Augmentation

To increase the overall number of training samples, it is crucial to apply several data augmentation strategies, such as flipping, rotation, or color jittering. A common data augmentation scheme for training images [15,16,17] is as follows: The image is resized with its smallest dimension randomly sampled between 256 and 480 for scale augmentation, then a 224 × 224 region is randomly cropped out, which is followed by a random horizontal flip, standard color augmentation, and per-pixel mean subtraction. At test time, a 224 × 224 center crop is applied, as in [17].

However, standard data augmentation strategies might not be suitable for recognizing nutrient deficiency, since they might discard import information that is necessary to identify the subtle differences of nutrient deficiency symptoms as shown in Figure 1. We therefore evaluated the impact of the data augmentation on the accuracy where we focused on cropping and padding with different scales. The results are shown in Table 4. For cropping, the smallest image side was firstly resized to a fixed scale *R*, and then a C×C region was randomly cropped (C∈[224,384,592,800]). This serves as a data augmentation scheme, because it increases the size of our training set by a factor of (R−C+1) × (R−C+1). We compare the cropping approach with a padding approach. In this case, the largest image side was resized to *C* and padding was used to fill the shorter image side in order to obtain a quadratic input image. We denote the second approach by ‘resize + padding’. All of the experiments were conducted with DenseNet-161.

Table 4 shows that a large difference between *R* and *C* results in a steep drop in accuracy, as it refers to a tiny patch of the original image that may lack discriminative patterns for nutrient deficiency detection. A higher resolution of the input image also contributes to the final prediction accuracy, as subtle symptoms cannot be recognized in low resolution images. However, the higher resolution is accompanied by a smaller batch size due to memory limitations and the performance deteriorates if the batch size becomes very small. The results show that a resolution of 592 × 592 pixels performs best for a GPU with 11 GB RAM. When we compare the cropping with the padding, we observe that padding performs better. Note that, in each row of Table 4, the input size is the same, e.g., 224 × 224 pixels for the first row.

We further evaluated the impact of other data augmentations strategies, including flipping, rotation, color jitter, and noise. We experimented with different amounts of both Gaussian and impulsive noise and report the best result using Gaussian noise with zero mean and standard deviation 0.01. The accuracy improves by introducing additional geometric transformations, like horizontal flipping or image rotation, as shown in Table 5. This is expected, since such geometric changes reflect viewpoint changes. In contrast, adding color jitter or noise decreases the accuracy, likely because these strategies intrinsically change the latent patterns that are subtle across different classes in this scenario. Finally, the combination of horizontal flipping and image rotation achieves the best performance with 98.4% top-1 accuracy.

### 6.3. Evaluation across Crop Development Stages

The crop development stage is a special property of DND-SB as compared to other datasets, because the dataset itself changes in a continually evolving way. Although the sample distributions remain the same under controlled conditions, the feature distributions may demonstrate significant variations over time as the plants grow. Besides, it is crucial to recognize the nutrient status of crops in a later stage with images collected before. We have adopted different dataset splits to evaluate this challenging property.

In Table 6, we used a single date as test set and trained DenseNet-161 with images from the other dates. We compare each result to a random dataset split with the same ratio of training images to test images. The dates corresponding to each number are provided in the first two rows of Table 1. Generally, the prediction results are relatively stable (97–99%) when all of the images were randomly sampled. However, if the test data are from a different crop development stage than the training samples, then the task becomes much more challenging. Intuitively, as growing crops are heavily affected by changing environmental conditions as well as by abiotic and biotic stresses, the feature distributions among crop development stages do not fulfill the i.i.d. condition, which is the fundamental assumption for machine learning algorithms. Furthermore, there is a large drop of accuracy when the test set consists of images from the last date, likely because the time interval between 9 and 10 (3 October 2019 to 4 November 2019) is larger when compared to the other time intervals. That is, crops apparently grew fast with clear visual variation in this period, so that a model trained with previous images struggled to anticipate their patterns at a later stage.

We also evaluated the accuracy when the training set only contained images whose dates of acquisition were earlier than the one in the test set. Most of the models failed to anticipate future patterns (with accuracy less than 50%), as shown in Table 7. However, an early detection of nutrient deficiencies based on images in early stages is crucial to enable timely actions like fertilization to prevent major losses or to increase potential yield. For this purpose, domain adaptation approaches should be applied to deal with this domain discrepancy problem caused by different crop development stages.

### 6.4. Qualitative Results

**Figure 7:**
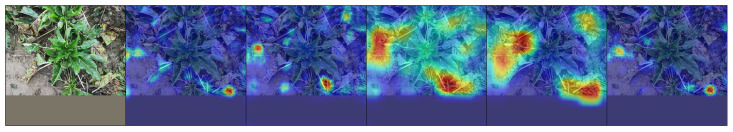
Saliency visualization of all network architectures. (column 1) original image. (column 2–6) Grad-CAM++ for AlexNet, VGG-16, ResNet-101, DenseNet-161, and SqueezeNet1_1, respectively. The ground truth label is NPK_.

**Figure 8:**
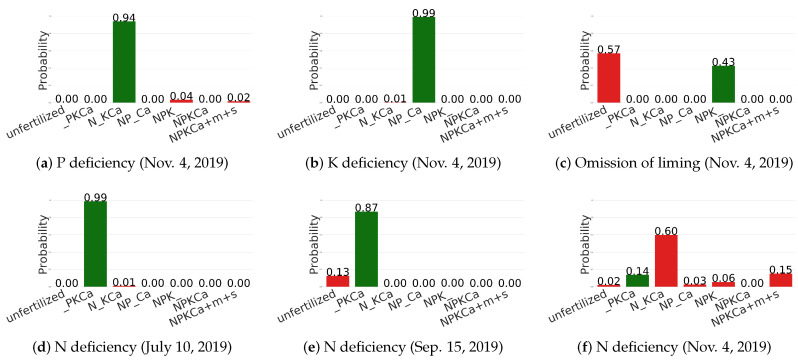
Histogram diagram of probability distribution over seven classes of nutrient treatments. Green bars indicate correct predictions while red bars indicate wrong predictions. The corresponding test images are shown in Figure 1.

In this section, we provide some qualitative results to provide deeper insights for these networks while classifying nutrient deficiencies in sugar beets. We extracted the learned features from the last CNN layer of each network and visualized them by utilizing Grad-CAM++ [41], a visualization tool that produces a coarse localization map highlighting the saliency regions in the image that have a high impact on the final prediction. Figure 7 shows the visualization results. ResNet-101 and DenseNet-161 output their predictions with visual attention on large patches containing older leaves, where manifested pathological symptoms caused by deficiencies of phloem mobile nutrients, such as N, P, and K, were obviously observed. However, the other three models only focus on local areas. We also observe that all of the models take the soil into account, which is a limitation of all networks and it is an important research direction to ensure that the networks only focus on the plants. We also report the predicted probabilities for each of the six images from Figure 1 while using the evaluation protocol of Table 6, i.e., we excluded all of the images that were taken at the same date for training. As we can see from Figure 8a,b, the P and K deficiency are correctly estimated, although the images look very similar (Figure 1a,b). However, the omission of liming is confused with the unfertilized treatment. As it was already observed in Table 6, the latest date (11/04) is, by far, the most difficult date. This is also reflected in Figure 8d–f, where the N deficiency is correctly estimated for the images in Figure 1d,e, but not for Figure 1f.

**Figure 9:**
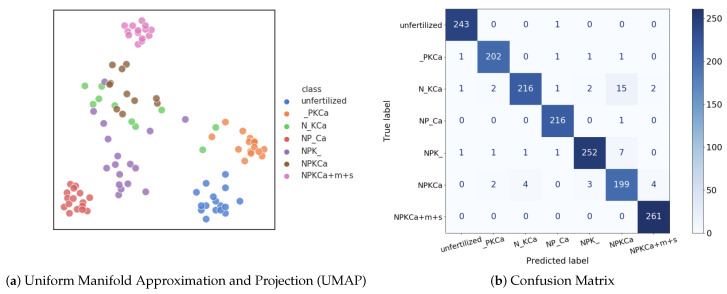
(**a**) UMAP result with the features extracted from DenseNet-161 before the last FC layer. 100 samples are randomly chosen for simplification. (**b**) Confusion matrix for DenseNet-161.

In order to visualize how well the different classes are separated in the feature space, we extracted the 2208-dimensional feature vectors before the last fully-connected layer of DenseNet-161 and utilized Uniform Manifold Approximation and Projection [42] (UMAP) for dimension reduction. UMAP is a nonlinear dimensionality reduction technique with better visualization quality when compared to other dimension reduction approaches [43], because it preserves more of the global structure. For better visualization, the original feature vectors were reduced to two dimensions, as shown in Figure 9a. The UMAP results illustrate distinct clusters of different categories, especially for unfertilized, _PKCa, NP_Ca and NPKCa+m+s treatments. The samples of the category N_KCa overlap slightly with the samples of the category NPKCa, so that they are more likely to be classified as NPKCa, and vice versa, which is also visible in the confusion matrix that is shown in Figure 9b. This is expected, since P deficiency is considered to represent the most difficult macro nutrient deficiency to be recognized in sugar beets [30].

## 7. Discussion

### 7.1. Dataset

Changing environmental conditions, as well as abiotic and biotic stresses that have occurred during the growth period, may affect the plants of one treatment more when compared to the plants of the other treatments. The growth period of 2019 was extraordinary dry and sugar beets showed severe drought stress symptoms in some weeks, mainly during 26 August 2019 and 3 September 2019. Besides, the soil conditions in different locations differ a lot and symptoms of nutrient deficiencies differ between crop species and even between crop cultivars. While the dataset is unique since it has been collected in the field and focuses on diagnosing different types of nutrient deficiencies in sugar beets, there is a need for more related datasets, such that data from different years, locations, crop species, and crop cultivars can be used to evaluate the robustness of methods for recognizing nutrient deficiencies. Nevertheless, the proposed dataset is already a very useful benchmark. In particular, the experimental setting where nutrient deficiencies need to be recognised across different crop development stages is a very challenging task.

### 7.2. Methodology

While very high recognition rates are achieved when the training data contains the same crop development stages as the test data, the CNNs trained with images of the plants in early stages struggle to anticipate the appearance of the plants in later stages, and vice versa. The generalization across different crop development stages is a current limitation of all deep learning approaches and it needs to be addressed as part of future work. Similarly, achieving good results across different locations, environmental factors, and crop cultivars is an open research problem. To this end, methods for domain adaptation [44] need to be investigated, which use an annotated training dataset and transfer the learned knowledge to a new dataset that is not or only partially annotated. The purpose of domain adaptation approaches is to bridge differences between training and test data such that differences of location, soil, weather conditions, and crop cultivars can be addressed.

P deficiency is, even for experts, the most difficult macro nutrient deficiency to be recognized in sugar beets [30]. We observed this also for our evaluated CNNs as shown in Figure 9. It remains an open question whether P deficiency can be recognized from RGB sensors alone or if other sensors need to be used and combined with RGB sensors. We also observed that the networks do not focus purely on the plants, but they take the soil into account as well. This results in a lack of robustness if the location changes and it is a relevant question how the networks can be enforced to focus exclusively on the plants. While segmenting the plants is an option, there might be better ways to deal with this issue. For example, visual attention mechanism [45,46,47] can be adopted to learn weighting masks that weight features of plants and soil by different levels of importance and, hence, discard irrelevant information that is not related to the plant. Related to this issue is the observation that training a CNN from scratch does not yield satisfactory results. The recent work [48] shows that ImageNet has a strong bias towards recognizing textures rather than shapes. However, the textures learned from unrelated categories are not plausible for agricultural purposes. Therefore, it is worth investigating whether pre-training the models on agricultural datasets to customize an agriculture-oriented prior knowledge would help.

## 8. Conclusions

Nutrient deficiencies represent major constraints for crop production worldwide and even common nutrient deficiencies, such as K and P deficiencies, can currently not be accurately diagnosed by non-invasive methods particularly in early stages. Likewise, non-invasive methods for diagnosing N deficiency are often inaccurate. In this paper, we proposed a new dataset consisting of RGB images that were taken from sugar beets that received seven types of nutrient treatments. The dataset is a unique benchmark since the crop was growing as part of long-term fertilizer experiments under real field conditions. As baseline, we evaluated five convolutional neural networks that provide different trade-offs between accuracy and processing speed. While the best network was able to recognize the symptoms of nutrient deficiencies in sugar beets with a high accuracy if all development stages have been observed during training, recognizing nutrient deficiencies across crop development stages remains an open problem. Moreover, the distraction of networks caused by the soil is still a limiting factor towards robust recognition of nutrient deficiency. Therefore, future work should focus on visual attention mechanisms, generalization, transferability to other sites or cultivars, and on the adaptation to changing environmental conditions.

## Figures and Tables

**Table 1:** The number of images in the Deep Nutrient Deficiency for Sugar Beet (DND-SB) dataset for the training and test set. **“_”** stands for the omission of the corresponding nutrient, **07/10** denotes 10 July 2019, where 2019 is omitted for simplification.

No.	1	2	3	4	5	6	7	8	9	10	
Class/Date	07/10	07/19	07/30	08/04	08/15	08/26	09/03	09/15	10/03	11/04	Total
unfertilized	85	56	50	111	97	119	59	70	145	76	868
_PKCa	86	56	/	105	64	111	72	69	55	90	708
N_KCa	63	61	66	116	89	124	49	71	79	90	808
NP_Ca	68	64	63	118	79	108	55	62	83	94	794
NPK_	79	61	57	119	100	105	52	67	153	100	893
NPKCa	58	53	/	118	71	117	79	90	/	90	676
NPKCa+m+s	75	54	/	128	86	131	77	86	172	92	901
total	514	405	236	815	586	815	443	515	687	632	5648

**Table 2:** Soil analysis data. Topsoil mineral N, plant available soil P and K ($P_{\mathrm{CAL}}$ and $K_{\mathrm{CAL}}$) extracted with a calcium-acetate-lactate extract in kg ha^−1^ and topsoil pH values for the seven treatments and four sampling dates in 2019 at the long-term fertilizer experiment Dikopshof. **07/10** denotes 10 July 2019, where 2019 is omitted for simplification.

Content	Sampling Date	Unfertilized	_PKCa	N_KCa	NP_Ca	NPK_	NPKCa
Mineral N	05/16	13.5	10.1	76.5	74.0	109.5	22.8
	06/13	20.7	5.2	18.8	26.6	36.2	9.4
	07/10	6.8	3.1	2.7	3.4	18.2	6.8
	09/10	3.2	1.8	4.0	8.1	3.6	4.1
$P_{\mathrm{CAL}}$	05/16	18	168	30	90	57	142
	06/13	35	182	36	88	67	158
	07/10	47	166	34	61	82	84
	09/10	18	147	28	91	65	147
$K_{\mathrm{CAL}}$	05/16	108	228	226	121	162	219
	06/13	126	243	198	100	144	211
	07/10	122	239	216	117	137	275
	09/10	76	320	285	84	117	462
pH value	05/16	5.8	6.9	6.4	6.6	5.5	6.5
	06/13	5.6	7.0	6.7	6.8	5.8	6.8
	07/10	5.5	6.9	6.4	6.8	5.8	6.3
	09/10	5.7	6.8	6.7	6.7	5.5	6.6

**Table 3:** Result table for different network architectures. DenseNet-161 outperforms other models in terms of test accuracy by a large margin, which shows its generalization ability due to higher parameter efficiency. The reported accuracy refers to the top-1 accuracy metric on the test set.

Model	Pre-Trained		From Scratch		Test Time (s/image)	Parameters (M)
	Accuracy (%)	Training Time (min)	Accuracy (%)	Training Time (min)		
random	14.3	-	14.3	-	-	-
AlexNet	87.4	27	62.4	41	0.009	57
VGG-16	89.7	105	66.8	466	0.046	134
ResNet-101	95.0	103	80.3	293	0.050	43
DenseNet-161	98.4	129	90.7	240	0.043	27
SqueezeNet	89.3	14	65.4	20	0.011	0.7

**Table 4:** Result table for cropping and padding.

Model	Resize (*R*)	Crop (*C*)	Accuracy (%)	Resize (*R*) + Padding	Accuracy (%)
DenseNet-161	256	224	82.4	224	86.1
DenseNet-161	480	224	64.5		
DenseNet-161	592	224	61.0		
DenseNet-161	416	384	90.1	384	91.5
DenseNet-161	624	592	93.2	592	95.8
DenseNet-161	832	800	89.1	800	76.4

**Table 5:** Ablation study of augmentation strategies.

Model	Flipping	Rotation	Color Jitter	Noise	Accuracy (%)
DenseNet-161	-	-	-	-	95.8
DenseNet-161	√	-	-	-	96.5
DenseNet-161	-	√	-	-	98.3
DenseNet-161	-	-	√	-	94.9
DenseNet-161	-	-	-	√	94.5
DenseNet-161	√	√	-	-	98.4
DenseNet-161	√	√	√	-	97.5
DenseNet-161	√	√	√	√	97.2

**Table 6:** **Left**: the dataset was split into training and test set according to the date of acquisition. In each experiment, images taken from a single date were chosen as test set while the others images as training set. **Right**: randomly sampled training and test sets. **#training:#test** denotes the ratio of the number of training images to the number of test images.

Training Set	Test Set	#training:#Test	Accuracy (%)	#Training:#Test	Accuracy (%)
				(Random)	(Random)
w/o 1	1	5134:514 (10:1)	74.7	5134:514 (10:1)	98.4
w/o 2	2	5243:405 (13:1)	84.4	5243:405 (13:1)	98.5
w/o 3	3	5412:236 (23:1)	80.9	5412:236 (23:1)	98.5
w/o 4	4	4833:815 (6:1)	78.4	4833:815 (6:1)	97.9
w/o 5	5	5062:586 (9:1)	80.6	5062:586 (9:1)	97.6
w/o 6	6	4833:815 (6:1)	76.8	4833:815 (6:1)	97.9
w/o 7	7	5205:443 (12:1)	78.3	5205:443 (12:1)	98.6
w/o 8	8	5133:515 (10:1)	78.8	5133:515 (10:1)	98.4
w/o 9	9	4961:687 (7:1)	80.1	4961:687 (7:1)	98.3
w/o 10	10	5016:632 (8:1)	48.5	5016:632 (8:1)	98.1

**Table 7:** **Left**: images in the training set were taken earlier than images in the test set. **Right**: randomly sampled training and test sets. **#training:#test** denotes the ratio of the number of training images to the number of test images.

Training Set	Test Set	#Training:#Test	Accuracy (%)	#Training:#Test	Accuracy (%)
				(Random)	(Random)
1-9	10	5016:632 (8:1)	48.5	5016:632 (8:1)	98.1
1-8	9-10	4329:1319 (3:1)	54.0	4329:1319 (3:1)	95.9
1-7	8-10	3814:1834 (2:1)	61.6	3814:1834 (2:1)	96.8
1-6	7-10	3371:2277 (1.5:1)	75.4	3371:2277 (1.5:1)	95.0
1-5	6-10	2556:3092 (0.8:1)	47.5	2556:3092 (0.8:1)	93.0
1-4	5-10	1970:3678 (0.5:1)	43.6	1970:3678 (0.5:1)	91.5
1-3	4-10	1155:4493 (0.26:1)	44.2	1155:4493 (0.26:1)	87.2
1-2	3-10	919:4729 (0.2:1)	41.5	919:4729 (0.2:1)	84.6
1	2-10	514:5134 (0.1:1)	31.8	514:5134 (0.1:1)	79.4
